# Th17-like cells and immunosuppressive macrophages infiltrate tertiary lymphoid structures with distinct maturation status in soft-tissue sarcoma

**DOI:** 10.1038/s41419-025-08376-4

**Published:** 2025-12-22

**Authors:** Giulia Petroni, Federico Scolari, Guido Scoccianti, Annarita Palomba, Daniela Greto, Simone Romagnoli, Ilaria Palchetti, Andrea Bernini, Enrico Caliman, Simone Polvani, Filippo Nozzoli, Beatrice Menicacci, Giulia Nannini, Domenico Andrea Campanacci, Serena Pillozzi, Lorenzo Antonuzzo

**Affiliations:** 1 Department of Experimental and Clinical Medicine, University of Florence, Florence, Italy; 2 Department of Experimental and Clinical Biomedical Sciences “Mario Serio”, University of Florence, Florence, Italy; 3 Department of Health Sciences, University of Florence, Florence, Italy; 4 Orthopaedic Oncology and Reconstructive Surgery Unit, Careggi University Hospital, Florence, Italy; 5 Histopathology and Molecular Diagnostics, Careggi University Hospital, Florence, Italy; 6 Radiotherapy Unit, Careggi University Hospital, Florence, Italy; 7 Department of Chemistry “Ugo Schiff”, University of Florence, Florence, Italy; 8 Department of Biotechnology, Chemistry and Pharmacy, University of Siena, Siena, Italy; 9 Oncology Unit, Careggi University Hospital, Florence, Italy

**Keywords:** Prognostic markers, Tumour immunology

## Abstract

Tertiary lymphoid structures (TLSs) have been associated with favorable clinical outcome and improved responses to immune checkpoint inhibitors in soft-tissue sarcomas (STSs). However, due to their rarity and high heterogeneity, information regarding the mechanisms involved in TLS formation and contribution to antitumor immunity in STS are extremely elusive. To address this gap, we integrated immunohistochemistry and transcriptomic analyses from 31 treatment-naïve STS specimens from an independent cohort of patients with primary or locally recurrent disease. Further validation was conducted using external bulk, single-cell and spatial transcriptomics data from 5 publicly available datasets. We found TLSs to be highly heterogeneous in terms of amount, localization, maturation status and cellular composition, and corroborated previous findings showing that their presence, along with B cells in the STS microenvironment, are good indicators of favorable prognosis. Transcriptomic analysis showed that high expression of germinal center (GC) B cell-related genes was associated with TLS presence and with an upregulation of signatures specific for T helper 17 (Th17) cells in STS and other cancer types. Conversely, genes signatures discriminating for immunosuppressive M2-like macrophages were enriched in tumors with low expression of GC B cell-related genes. Immunohistochemistry showed distinct spatial patterns for Th17-like cells and M2-like macrophages within TLS areas, with IL17A^+^ cells predominantly localized within intratumoral mature TLSs, and CD163^+^ macrophages mainly observed in immature TLSs. Integrating these findings, we identified tumors with high expression of GC B cell- and Th17 cell-related signatures together with low fractions of M2-like macrophages, to have superior survival outcomes. Herein, our findings point out to two cellular players (Th17-like cells and M2-like macrophages) with potentially opposite roles in STS-associated TLS formation and maturation, thus providing the basis for future research efforts aiming at the development of therapeutic immunological interventions to enhance TLS-mediated antitumor immunity in STS.

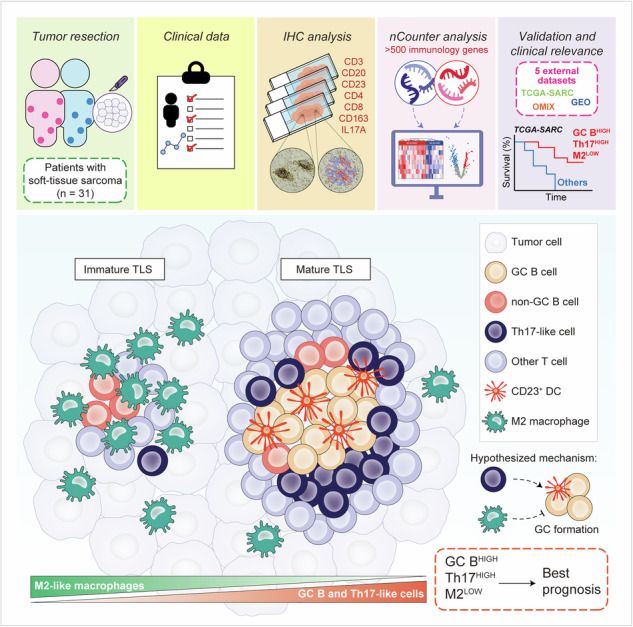

## Introduction

Soft-tissue sarcomas (STSs) are rare and highly heterogeneous tumors of mesenchymal origin. According to the 2020 WHO classification, STSs comprise approximately 70 histological and molecular subtypes characterized by distinct morphology and diverse clinical behavior, thus making STSs challenging tumors not only to diagnose but also to treat [1]. Recently, numerous ongoing trials have been designed to investigate the efficacy of inhibitors of PD-1 or of its ligand (PD-L1), both as single agents and as combination regimens, in patients with advanced STS (ClinicalTrials.gov). However, results reported so far are unsatisfactory. Indeed, objective response rates (ORR) varied among STS patients with different subtypes, ranging from 6 to 18% for patients with dedifferentiated liposarcoma and from 20 to 40% for undifferentiated pleomorphic sarcoma, while no benefit has been observed for leiomyosarcoma patients [2, 3]. Moreover, while only modest additional clinical benefits have been shown in studies with immunomodulatory combination strategies [4, 5], the recent SU2C-SARC032 trial demonstrated a significant improvement in disease-free survival with the addition of neoadjuvant and adjuvant pembrolizumab to preoperative radiotherapy and surgery for stage III STS patients [6].

Interestingly, the retrospective transcriptomic profiling of STS specimens has identified signatures specific for tertiary lymphoid structures (TLSs) and B cells as positive predictive biomarkers of response to immune checkpoint inhibitors (ICIs) in these patients [7, 8]. In addition, increased ORR and progression-free survival (PFS) have been observed in patients with STS characterized by the presence of a TLS-related signature in baseline specimens (ORR: 30%; median PFS: 4.9 months), compared to the overall STS population (ORR: 2%; median PFS: 1.5 months), in a non-randomized phase 2 clinical trial [9].

TLSs are considered tumor-associated privileged “niches” where both humoral and cell-mediated immune responses can be generated or boosted. They can vary in their composition, location, density and maturation status, depending on the cancer type. Bona fide, mature TLS (mTLS) are composed of CD20^+^ B cells forming a core germinal center (GC), adjacent to CD8^+^ effector T cells, CD4^+^ follicular helper T (Tfh) cells, follicular dendritic cells (DCs) and antibody-secreting plasma cells [10]. Noteworthy, growing preclinical and clinical studies are rapidly improving our understanding on the TLS immunobiology, and some therapeutic interventions have been proposed to enhance their formation and effector functions in some settings [11]. However, information regarding mechanisms at the basis of TLS development and involvement in naïve and treatment-mediated antitumor immunity in STS are extremely elusive, thus limiting the identification of therapeutic strategies that could be used to improve STS survival outcomes.

Here, we employed immunohistochemistry (IHC) alongside transcriptomics to characterize the immunological contexture of STS-associated TLSs and found that TLS maturation is associated with the presence of T helper 17 (Th17)-like cells inside TLS areas, but reduced TLS infiltration by CD163^+^ M2-like macrophages. Furthermore, integrating gene expression signatures for GC B cells, Th17 cells and immunosuppressive M2-like macrophages we refined a novel TLS-related immunological signature that accurately predicts STS survival outcomes.

## Materials/Subjects and methods

### Patient population

This study includes 31 adult patients with primary or locally recurrent STS treated and followed-up at the Department of Oncological and Reconstructive Orthopaedics of the Careggi University Hospital (Florence, Italy), between October 2020 and August 2022. The study was approved by the Ethics Committee of Tuscany (Italy), Comitato Etico Regionale per la Sperimentazione Clinica della Regione Toscana Sezione AREA VASTA CENTRO (protocol n.16933_bio), and conducted in accordance with the ethical principles included in the Declaration of Helsinki. Written informed consent was provided by all study participants. All pathological diagnoses of STS were confirmed by the Histopathology and Molecular Diagnostic Unit, Careggi University Hospital (Florence, Italy). For each patient, the most suitable treatment approach was planned during the multidisciplinary team meeting. All patients underwent surgery with curative intent. The following data were collected from each patient’s medical record: clinical-demographic characteristics at diagnosis, data about preoperative and postoperative treatments received by patients, type of surgery, histopathological diagnosis of the surgical sample, the time and type of any tumor recurrence (local or distant) and whether it occurred. The clinical outcomes evaluated comprised: relapse-free survival (RFS), defined as the time elapsed from the date of curative surgery to the time of recurrence or death, and overall survival (OS), defined as the time measured from surgery until death of patient. The cut-off date for the follow-up analysis was June 01, 2024. Clinicopathological information of all patients are listed in Table 1.

**Table 1:** Patient characteristics.

	All	Relapse	No Relapse		
Characteristics	*n* = 31	*n* = 12	*n* = 19	*P* value	Statistic
Age, median (range)	68 (38-92)	76 (38-87)	67 (38-92)	0.264	Wilcoxon rank-sum test
Gender, *n* (%)	–	–	–	0.149	Fisher’s exact test
Male	15 (48.4%)	8 (66.7%)	7 (36.8%)	–	–
Female	16 (51.6%)	4 (33.3%)	12 (63.2%)	–	–
Anatomical site of primary tumor, *n* (%)	––	–	–	0.431	Fisher’s exact test
Trunk	2 (6.5%)	0 (0%)	2 (10.5%)	–	–
Upper extremity	5 (16.1%)	3 (25.0%)	2 (10.5%)	–	–
Lower extremity	24 (77.4%)	9 (75.0%)	15 (78.9%)	–	–
Primary *vs* locally recurrent STS, *n* (%)	–	–	–	0.630	Fisher’s exact test
Primary tumor	27 (87.1%)	10 (83.3%)	17 (89.5%)	–	–
Locally recurrent tumor	4 (12.9%)	2 (16.7%)	2 (10.5%)	–	–
Type of surgery, *n* (%)	–	–	–	0.611	Fisher’s exact test
Wide resection	26 (86.7%)	9 (81.8%)	17 (89.5%)	–	–
Marginal resection	4 (13.3%)	2 (18.2%)	2 (10.5%)	–	–
N/A	1 (3.2%)	1 (8.3%)	0 (0%)		
Subtype, *n* (%)	–	–	–	0.057	Fisher’s exact test
Pleomorphic sarcoma	15 (48.4%)	5 (41.7%)	10 (52.6%)	–	–
Liposarcoma	9 (29.0%)	2 (16.7%)	7 (36.8%)	–	–
Myxofibrosarcoma	6 (19.4%)	5 (41.7%)	1 (5.3%)	–	–
Leiomyosarcoma	1 (3.2%)	0 (0%)	1 (5.3%)	–	–
Grade, *n* (%)	–	–	–	0.510	Fisher’s exact test
Low (G1–G2)	2 (6.5%)	0 (0%)	2 (10.5%)	–	–
High (G3)	29 (93.5%)	12 (100%)	17 (89.5%)	–	–
Pre- or postoperative treatment *n* (%)	–	–	–	0.329	Fisher’s exact test
Preoperative RT	4 (12.9%)	0 (0%)	4 (21.1%)	–	–
Preoperative CT–RT	1 (3.2%)	0 (0%)	1 (5.3%)	–	–
Postoperative RT	12 (38.7%)	6 (50.0%)	6 (31.6%)	–	–
Postoperative CT	1 (3.2%)	0 (0%)	1 (5.3%)	–	–
Postoperative CT–RT	1 (3.2%)	1 (8.3%)	0 (0%)	–	–
None	12 (38.7%)	5 (41.7%)	7 (36.8%)	–	–

*CT* chemotherapy, *G* grade, *N/A* not available, *RT* radiotherapy, *STS* soft-tissue sarcoma.

### IHC staining

IHC stainings were performed on available treatment-naïve formalin-fixed paraffin-embedded (FFPE) 3 µm tissue sections (23 out of 31 patients from our cohort). For single IHC stainings with anti-CD3 (2GV6; #790-4341), anti-CD4 (SP35; #790-4423), anti-CD8 (SP57; #790-4460), anti-CD20 (L26; #760-2531), anti-CD163 (MRQ-26; #760-4437), and anti-CD23 (SP23; #790-4408) obtained by Ventana/Roche, sample processing was performed on consecutive sections using the automated immunostainer Ventana Discovery ULTRA platform (Ventana; Roche Diagnostics). Sections were deparaffinized in EZ prep (#950-102), antigen retrieval was achieved by incubation with cell-conditioning solution 1 (#950-124), and signals were developed with the UltraMap Red anti-mouse (#760-154) or anti-rabbit (#760-153) detection kits by Ventana/Roche, according to the manufacturer’s instructions. Immunostaining for IL17A was performed using the Bond-MAX automated immunostainer (Leica Biosystem) with Bond Polymer Refine Detection (#DS9800) after a deparaffinization step in Bond Dewax Solution (#AR9222) and antigen retrieval with Bond Epitope Retrieval Solution 1 (#AR9961), all obtained by Leica Biosystem. Anti-IL17A antibody (#ab79056; Abcam) was used at 1:200 dilution.

### Quantitative assessment of TLSs

IHC sections were scanned using the Aperio LV1 real-time digital pathology system (vLV1; Leica Biosystem). TLSs were quantified in digitalized whole-tissue slides based on CD20/CD3 staining, as previously described [12]. Only TLSs made up of more than 50 cells were included in the analysis, and TLSs were defined as mature when at least one CD23^+^ cell displaying dendritic morphology features was detected within the TLS area, as previously described [12]. Quantitative analysis was independently performed by 2 investigators and reviewed by 1 expert pathologist. Tissue area, TLS area and TLS distance from the tumor border (TB) were measured by the Aperio ImageScope software (v12.4.6.5003; Leica Biosystem). TLS area was delineated on both specimens stained for CD3 and CD20, and the larger area was selected for quantitative analyses. Total TLS density was calculated for each slide as the number of total TLSs *per* total tissue area (considering only the tumor area plus the peritumoral tissue within 1 mm of the TB).

### Assessment of cellular composition

Cell densities (cells/mm^2^) for CD4^+^ T cells, CD8^+^ T cells, CD20^+^ B cells, CD23^+^ DCs, and CD163^+^ macrophages in each sample were calculated in TLS areas and in the entire tissue section by the QuPath software (v0.5.1; https://qupath.github.io/) using the “positive cell detection” algorithm. Thresholds were manually adjusted for each type of staining and cell detection was visually verified to have occurred correctly. The spatial distribution of each cell population was determined by dividing whole-tissue slides in 3 regions: 2 intratumoral regions, further divided in tumor center and inner invasive margin (IIM; within 1 mm of the TB), and 1 peritumoral region corresponding only to the healthy tissue within 1 mm of the tumor front (also referred as to outer invasive margin; OIM). Cell densities in the entire tumor microenvironment (TME) were determined for 4-to-8 round ROIs (area: 1 mm^2^) for each of the above-mentioned regions and for a representative number of TLSs. Necrotic and ulcerated areas were excluded from the analysis.

### Evaluation of IL17A staining

A TLS was considered positive for IL17A staining when at least one cell forming the TLS exhibited positive reaction for IL17A staining together with lymphoid morphological features. A total of 123 TLSs from 10 out of 13 TLS^+^ samples were included in this analysis. TLSs surrounded by a high background were excluded to avoid false positives. Challenging cases were confirmed by assessing the concordance between IL17A and CD4 positivity on single IHC carried out on adjacent FFPE 3 µm tissue sections. IL17A expression by tumor cells was evaluated semi quantitatively and scored considering both the percentage of IL17A-positive (IL17A^+^) cells and staining intensity. Percentage of positively stained cells was scored as: no cells with positive reaction, 0; <10% cells with positive reaction, 1; 10–50% cells with positive reaction, 2; 51–80% cells with positive reaction, 3; >80% cells with positive reaction, 4. Intensity was evaluated as: no staining, 0; weak staining, 1; moderate staining, 2; strong staining, 3. The final score is the result of multiplying these values. IHC stained slides for IL17A positivity were independently evaluated by two pathologists and challenging cases were discussed under the microscope until full consensus was achieved.

### nCounter NanoString gene expression profiling

STS specimens were processed for nCounter NanoString gene expression profiling. Total RNA was retrieved from 15-30 mg frozen tumor samples using the RNeasy Mini kit (#74104; Qiagen) after disruption and homogenization with a TissueLyser (Qiagen). When frozen samples were not available, RNA isolation was performed using 1-to-2 15 µm-thick FFPE tissue sections and the RNeasy FFPE kit (#73504; Qiagen). RNA concentration and quality were checked using the NanoDrop 2000 (Thermofisher). Gene expression was evaluated using the nCounter Human Immunology v2 panel (#XT-CSO-HIM2-12; NanoString Technologies), according to the manufacturer’s instructions.

nCounter data were normalized using the Remove Unwanted Variation (RUV) method adapted for nCounter data [13], with a single dimension of unwanted variation and the default housekeeping genes indicated in the Immunology v2 panel. Differential gene expression analysis (DGEA) was performed using DESeq2 v1.44.0 on raw counts [14], as previously described [13]. Genes were considered differentially expressed with an adjusted *P* value < 0.05 and [log_2_(fold change)] >0.5.

### Processing of public transcriptomic datasets

For bulk data processing, TCGA-SARC primary tumor RNA-sequencing (RNA-seq) data were downloaded from GDC using TCGAbiolinks v2.32.0 package. Genes with counts = 0 in more than half of the total samples were removed. Raw counts were normalized using the trimmed mean of M-values method and log2-transformed. Samples with missing clinical data were excluded from further analysis. GSE213065 normalized transcriptomic data and GSE272346 raw counts were downloaded from the GEO portal. GSE272346 data were normalized and log-transformed. DGEA was performed on raw TCGA-SARC counts using DESeq2. To reduce background noise in DEG identification, genes with <10 counts across all samples were excluded from the analysis. Gene set enrichment analysis (GSEA) was performed using clusterProfiler v4.12.6 package with the fgsea method [15]. Genes were ranked by the Wald statistic from the DGEA results and enrichment was conducted on Gene Ontology (GO) Biological Processes. Enrichment results were plotted using enrichplot v 1.24.4 package. Relative cell fractions have been calculated on TCGA-SARC and GSE272346 counts per million and on GSE213065 transcripts per million using CIBERSORT with the LM22 signature matrix [16].

For single-cell data analyses, OMIX009480 raw matrices were loaded and preprocessed using scanpy (v1.11.4) [17]. Doublets were detected with Scrublet (v0.2.3) and cells with doublet score above 0.2 were removed [18]. Cells with fewer than 200 or more than 4,000 genes, over 20% mitochondrial transcripts, or over 25.000 total counts were filtered out, and genes expressed in less than 3 cells were also excluded. Filtered samples were normalized, log-transformed and scaled after regressing out the effects of total counts and mitochondrial content. CD45⁺ cells were identified based on PTPRC expression and pseudobulk expression profiles were generated. Differential expression between TLS phenotypes on pseudobulk counts was performed in R using DESeq2. Genes were ranked by Wald statistics for GSEA, which was conducted on GC B, Th17 and M2 macrophage signatures.

For GeoMx data analyses, GSE289272 DCC files, probe assay metadata (PKC) and annotation files were downloaded from the GEO portal and used as input for the R package GeomxTools [19]. The region-of-interests (ROIs) were classified as chronic nasopharyngitis or nasopharyngeal carcinoma (NPC) tissues. For downstream analysis, only NPC ROIs were retained. The pre-processing was made by several steps, including quality control (QC), filtering, normalization and dimensionality reduction. The QC assed sequencing quality for every ROI segment based on the following parameters: a minimum of 1000 reads per segment, 80% trimmed reads, 80% stitched reads, 80% aligned reads, a minimum of 10 negative counts, a maximum of 9000 counts in the NTC well, a minimum of 100 estimated nuclei. Quartile 3 (Q3) normalization was applied to standardize the dataset. The normalized dataset served as input for Gene Set Variation Analysis (GSVA) to compute the scores of the signatures of interest.

### Clustering

nCounter and TCGA-SARC samples were clustered based on the expression of GC B cells signature genes. Normalized log-scaled gene expression was used to calculate a Euclidean distance matrix, which was then used for hierarchical clustering with the Ward D2 method. The resulting dendrogram was cut into high- and low-expressing groups. Heatmaps were generated with the package ComplexHeatmap v2.13.1 using the scaled normalized gene expression.

Sarcoma immune classes (SICs) have been identified using the methods previously described [7].

### Cell signatures

Single-gene signatures were derived by dichotomizing the normalized log-scaled expression of the signature gene at the median value. Multiple-gene signatures were calculated dichotomizing the signature score at the median value. Signature scores were computed as the average of the normalized log-scaled counts of the genes in the signature. The M2 CIBERSORT signature was calculated by dichotomizing the M2 Macrophages relative cell fraction obtained in the deconvolution. Correlation between signature scores was evaluated with Pearson’s correlation. Differences in signature scores between groups were tested with a Wilcoxon rank-sum test. Multiple testing was addressed using the BH P-value adjustment method.

Gene signatures used in the nCounter cohort are reported in Table S1. Additional literature-based gene signatures were: 12-Chemokines signature [20], TLS signatures [21–24], M2 signature [25], and the GO signatures GO:0002314, GO:0042113, GO:0002467, GO:0072539, GO:0072540 and GO:0032740 (Ref. [26]).

The integrated signature between GC B cells, Th17 cells and M2 macrophages relative cell fraction was computed by averaging GC B and Th17 cells scores, then dichotomizing the resulting score at the median value. M2 cell fraction has been dichotomized at the median. Grouping was determined by selecting samples with high GC B/Th17 score and low M2 macrophage fraction versus all other samples.

### Statistical analysis

All statistical analyses and graphing were performed using R (v4.2) or GraphPad (v9.5.1). Clinical and demographic data were reported as median and range for continuous data and as number and percentage for categorical data. Comparisons were conducted using Wilcoxon rank-sum tests for continuous data and Fisher’s exact tests for categorical data. Multiple comparisons between continuous data were performed with Kruskal-Wallis followed by uncorrected Dunn’s test. Levene tests and Shapiro-Wilk tests were used to determine equality of variance and normality, respectively. Spearman’s rank correlation was estimated to determine the linear association between TLS density, TLS area and cell densities in the entire TME or in TLS areas. Unless specified, all tests are to be considered two-tailed.

Survival data were represented using a Kaplan-Mayer estimator, *P* values were calculated using a log-rank test. Univariate and multivariate analyses were performed using a Cox proportional hazard model. Only covariates significant in the univariable model were included in the multivariable model. Survival analyses were conducted using the survival v3.8 and survminer v0.5.0 packages. *P* values were corrected for multiple comparisons using the BH correction method unless otherwise stated. Significance level was set at *P* value < 0.05.

### Ethics approval and consent to participate

The study was reviewed and approved by the Ethics Committee of Tuscany (Italy), Comitato Etico Regionale per la Sperimentazione Clinica della Regione Toscana Sezione AREA VASTA CENTRO (Protocol n.16933_bio). The study was performed in accordance with the principles set by the Declaration of Helsinki. All patients signed written informed consent for treatmnent and study inclusion.

## Results

### Distribution pattern and clinical implication of TLSs in STS

For this study, treatment-naïve tumor specimens were collected from a total of 31 patients with localized primary or recurrent STS of the extremities and the trunk who underwent surgical resection with curative intent. Noteworthy, by categorizing patients in 2 groups based on disease recurrence after surgical resection of the tumor, no significant differences were observed for all the clinicopathological features, including sex and histotypes, among patients who eventually relapsed and non-relapsed patients (Table 1).

**Fig. 1:**
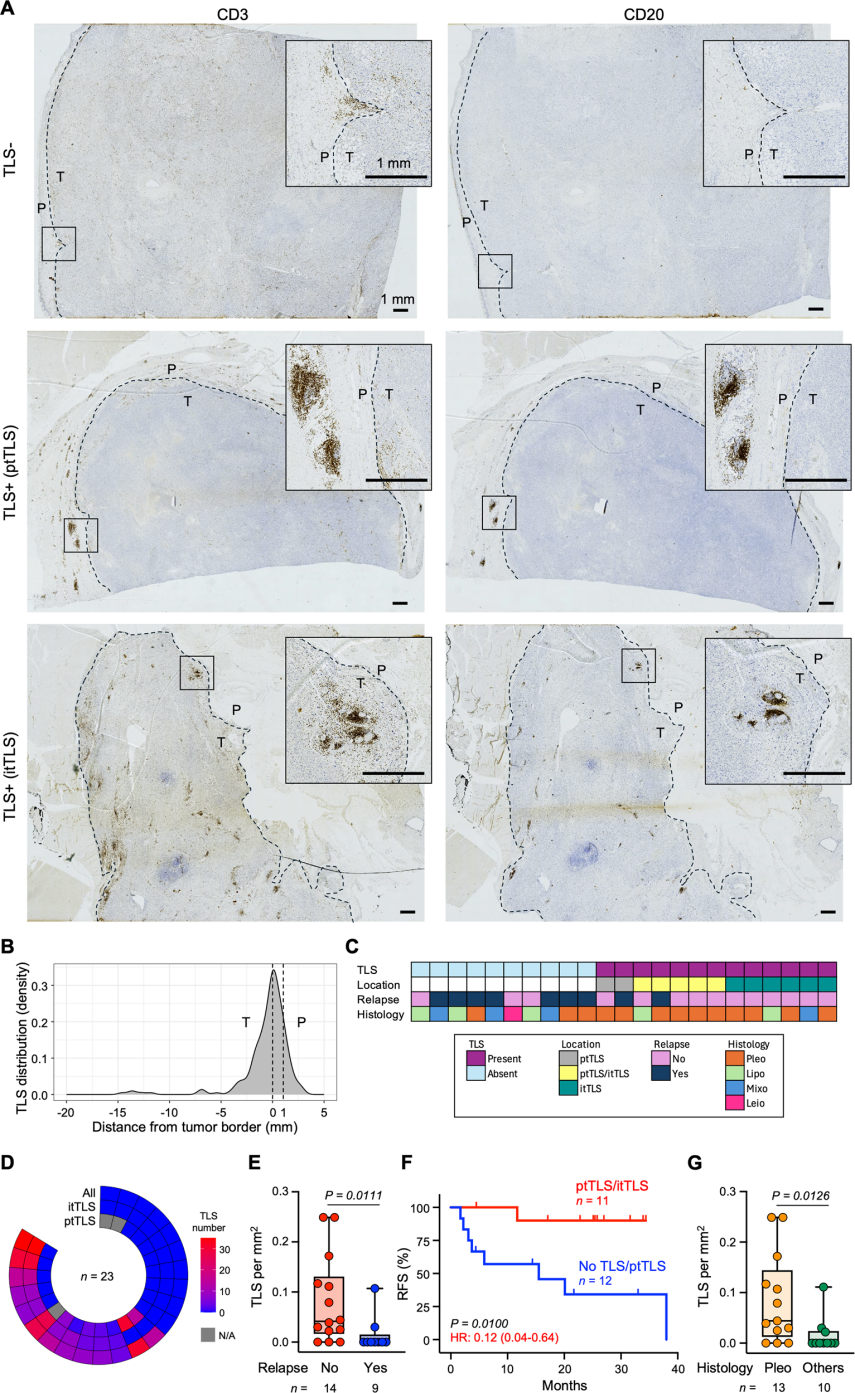
TLS distribution and clinical implication in STS. **A** Representative images for CD3 and CD20 expression in intratumoral (it) and peritumoral (pt) TLSs. Dotted lines show the tumor border. P, peritumoral healthy tissue; T, tumor. Scale bars: 1 mm. **B** Density distribution plot showing the distance of each TLS from the tumor border (*n* = 214 TLSs from 12 out of 13 TLS^+^ samples, as one TLS^+^ sample had no healthy tissue available for the analysis). Dotted lines show the tumor border (at 0) and the limit considered for the following analyses in the P area (1 mm range from the tumor border). **C** Associations between TLS presence, TLS location, relapse status evaluated as local or distant disease recurrence observed after surgical resection, and histological subtypes (Leio, leiomiosarcoma; Lipo, liposarcoma; Myxo, mixofibrosarcoma; Pleo, pleomorphic sarcoma) in 23 STS patients. **D** Distribution of ptTLS and itTLSs across STS specimens. Samples’ order is the same shown in Figs. 1C, 2C and S1D, 2D. **E**, **G** Box plot showing TLS density (count/mm^2^) in non-relapsed and relapsed patients (**E**), and in patients with Pleo or other STS histotypes (**G**). Each dot denotes one sample. Boxes show the median ± interquartile range; whiskers indicate minima and maxima. **F** Kaplan-Meier curve of relapse-free survival (RFS) among STS patients harboring at least one itTLS (ptTLS/itTLS group) and patients with no TLS or with only ptTLSs (no TLS/ptTLS group). Hazard Ratio (HR) and 95% confidence interval (calculated by log-rank method) are reported. *n* values represent the number of individual tumors or patients (**D**–**G**)*. P* values were calculated using an unpaired Mann-Whitney test (**E**, **G**) and log-rank test (**F**).

We initially evaluated the abundance and location of TLSs in adjacent surgical sections of tumor samples for 23 out of 31 STS patients enrolled, who did not receive preoperative radiotherapy (RT) nor chemotherapy (CT), by IHC. TLSs were defined as lymphoid aggregates of at least 50 immune cells, composed of both CD20^+^ B and CD3^+^ T lymphocytes in variable amounts (Fig. 1A), as previously described [12]. As both tumor and surrounding healthy tissues were available for most of the samples (20/23, 86.9%), TLS presence was initially evaluated in the whole slide (Fig. 1A). Location and number of TLSs were highly heterogeneous across STS samples. A total of 241 TLSs were identified in 13 samples (56.5%) ranging from a minimum of 4 to a maximum of 43 TLSs per slide (mean ± SEM number of TLS: 18.5 ± 3.4) (Table S2). Interestingly, TLS distribution peaked around the tumor border (TB) separating the malignant cell nest from the adjacent non-tumor tissue, with the majority (86.7%) of TLSs distributed intratumorally or peritumorally within a 1 mm range from the TB. Conversely, only a minority of peritumoral TLSs (ptTLSs; 13.3.%) was found between 1 to 3 mm and no TLSs were detected beyond 3 mm from the TB (Fig. 1B and Table S2), even in cases when more healthy tissue was available. Thus, for the following analyses we only considered ptTLSs located within a 1 mm range from TB (also referred to as OIM) and intratumoral TLSs (itTLSs), as previously described [7, 12]. TLS incidence was confirmed in 13 out of 23 samples (56.5%). itTLSs were found in 11/13 (84.6%) while ptTLSs in 7 out of 12 TLS^+^ (58.3%) samples with available peritumoral tissues (1 TLS^+^ sample and 2 TLS^-^ samples had no peritumoral tissue available for ptTLS evaluation) (Fig. 1C, D and Table S2). The presence of TLSs in one compartment was not exclusive to the other, as 5 samples harbored both itTLSs and ptTLSs in different ratios (Fig. 1C, D).

We next evaluated the prognostic implication of TLSs in our cohort of STS patients. Confirming the positive prognostic impact of TLSs in STS [7], TLS incidence, density and relative area were significantly higher in patients with no relapse (11/14, 78.6%) compared to patients who eventually relapsed (2/9, 22.%; *P* = 0.0131, Fisher’s exact test) (Fig. 1C, E and S1A). In addition, the presence of at least one itTLS was found to be better correlated with improved RFS - but not OS - than no TLS or TLS only located in the peritumoral area (Figs. 1F and S1B). We finally explored the association between TLSs and clinicopathologic characteristics. A higher TLS incidence (10/13 *vs* 3/10; *P* = 0.0397, Fisher’s exact test) and density were observed in pleomorphic sarcomas compared to other STS histotypes (Fig. 1C, G), which may explain their higher sensitivity to treatment with ICIs [2, 3], even though patients with pleomorphic sarcomas showed comparable survival outcomes to patients with other STS histotypes in our cohort (Figure S1C).

Overall, these results reveal a highly heterogeneous distribution pattern for STS-associated TLSs and confirm their prognostic value in STS.

### TLS maturation in STS is highly heterogeneous

**Fig. 2:**
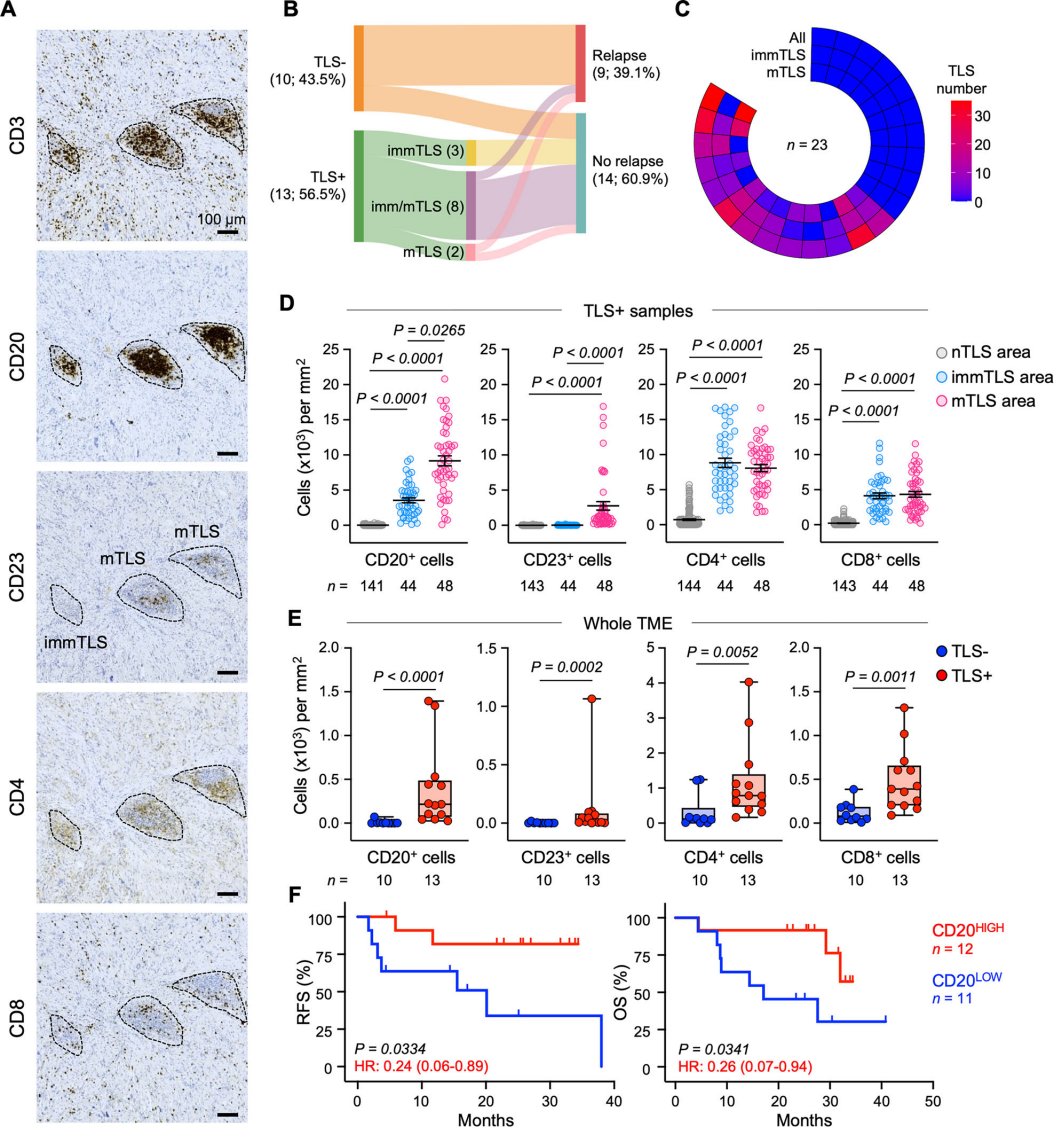
Cellular content of TLSs with distinct maturation status and of the entire STS TME. **A** Representative images of CD3, CD20, CD23, CD4 and CD8 expression in mature (m) and immature (imm) TLSs. Scale bars: 100 µm. **B** Sankey diagram showing the associations between TLS presence, TLS maturity and relapse status in 23 STS patients. **C** Distribution of mTLS and immTLS across STS patients. Samples’ order is the same shown in Figs. 1C, D, S1D and S2D. Density (count/mm^2^) of CD4^+^ cells, CD8^+^ cells, CD20^+^ cells and CD23^+^ cells in non-TLS (nTLS) and TLS areas (**D**), and in the whole TME (**E**), determined by IHC and QuPath analysis. In (**D**) each dot denotes one individual immTLS, mTLS or round ROI from nTLS area obtained from *n* = 13 TLS^+^ samples. Mean ± SEM is shown. In (**E**) each dot denotes one tissue sample, and values are expressed as the mean of 12-to-24 round ROIs from 3 tissue regions equally divided as follow: 2 intratumoral regions, further divided in tumor center and inner invasive margin (within 1 mm from the tumor front), and 1 peritumoral region corresponding only to the tissue surrounding the tumor nest within 1 mm from the tumor border (referred as to outer invasive margin). Boxes show the median ± interquartile range; whiskers indicate minima and maxima. **F** Kaplan-Meier survival curves for STS patients’ RFS and overall survival (OS) based on median CD20^+^ B cell density in the whole TME in high- ( ≥ median) and low-expressing groups (<median). HR and 95% confidence interval calculated by log-rank method are reported. *n* values represent the number of individual ROI/TLS (**D**), tumors (**C**, **E**) or patients (**B**, **F**)*. P* values were calculated using Kruskal-Wallis with uncorrected Dunn’s test (**D**), unpaired Mann-Whitney test (**E**), and log-rank test (**F**). In (**D**) only significant ( < 0.05) *P* values are shown.

Since TLS maturation is considered a key feature for supporting antitumor immunity and the presence of mTLS has been associated with improved prognosis and better response to immunotherapy in some cancer settings [12, 27–29], the maturation status of each TLS was then evaluated by IHC. Following a protocol validated on more than 350 tumor specimens, including 146 STSs [12], TLSs with at least one CD23^+^ cell displaying dendritic morphology features were defined as mTLSs (Fig. 2A). Interestingly, most TLS^+^ samples harbored both mature and immature TLSs (immTLSs; 8/13, 61.5%) in different proportions, with mTLS detected in 10 (76.9%) and immTLS in 11 out of 13 samples (84.6%) (Fig. 2B, C). Intriguingly, distribution of mTLS and immTLS was highly heterogeneous within intra e peritumoral compartments across STS samples (Figure S1D).

Together, these findings suggest that TLS maturity has no location specificity in STS and emphasize that maturation status contributes with location to the high intra-tumoral and inter-patient heterogeneity of TLS in STS. Noteworthy, patients harboring at least one mTLS showed improved RFS compared to patients with no TLS, but not compared to patients with noTLS/immTLS or with only immTLS (Figure S1E), as the latter group showed no relapse events (Fig. 2B). However, due to the limited number of samples included in the analysis, these findings require careful interpretation and further validation in larger datasets.

### Spatial distribution and clinical implication of B cells in STS

To gain a more comprehensive understanding of TLS heterogeneity (as well as of the entire TME) in STS, we next investigated the abundance of CD20^+^ B cells, CD23^+^ DCs, CD4^+^ T and CD8^+^ T cells by IHC (Fig. 2A). We first focused our analysis on the cellular composition of TLSs with different maturation status. As expected, mTLS areas showed a denser accumulation of CD20^+^ B cells and CD23^+^ DCs, as compared to immTLSs (Fig. 2D). However, this effect was not correlated to increased CD4^+^ and CD8^+^ T cell densities in mTLS (Fig. 2D). Interestingly, comparison of cellular densities in TLS areas *vs* non-TLS areas revealed a denser accumulation of all four cell populations inside TLSs (Fig. 2D), suggesting that, when these cells infiltrate STSs, they predominantly form lymphoid aggregates. As a direct consequence, when we looked at the whole TME (*i.e*., tumor plus OIM) the density of all four cell populations was significantly higher in TLS^+^ samples compared to TLS^-^ samples (Fig. 2E). Among these, T cell populations were the most abundant and mostly located at the IIM (*i.e*., the tumor area distant less than 1 mm from the border) (Figure S2A, B). However, although all cells positively correlated to TLS density and area, CD20^+^ B cell accumulation within the TME showed the strongest correlation, and a high infiltration of CD20^+^ B cells in the STS TME was significantly associated to favorable prognosis, considered as both RFS and OS (Fig. 2F and S2C–E). Of note, while also a higher accumulation of CD23^+^ cells in the TME of STS was significantly associated with prolonged RFS (but not OS), potentially due to its high correlation with CD20^+^ B cell density, the same results were not obtained upon stratification of patients based on median CD4^+^ and CD8^+^ T cell densities (Figure S2C–E).

Overall, these findings unravel that B cells infiltrating the sarcoma TME mostly reside in TLSs and confirm the clinical implication of B cells in STS, as already previously reported [7]. Thus, as B cells represent a key hallmark of STS-associated TLSs, their investigation represents a valid alternative to the detection of tumor-associated TLSs to predict STS prognosis.

### Identification of a 7-gene signature and its association with TLS presence and patient outcomes

**Fig. 3:**
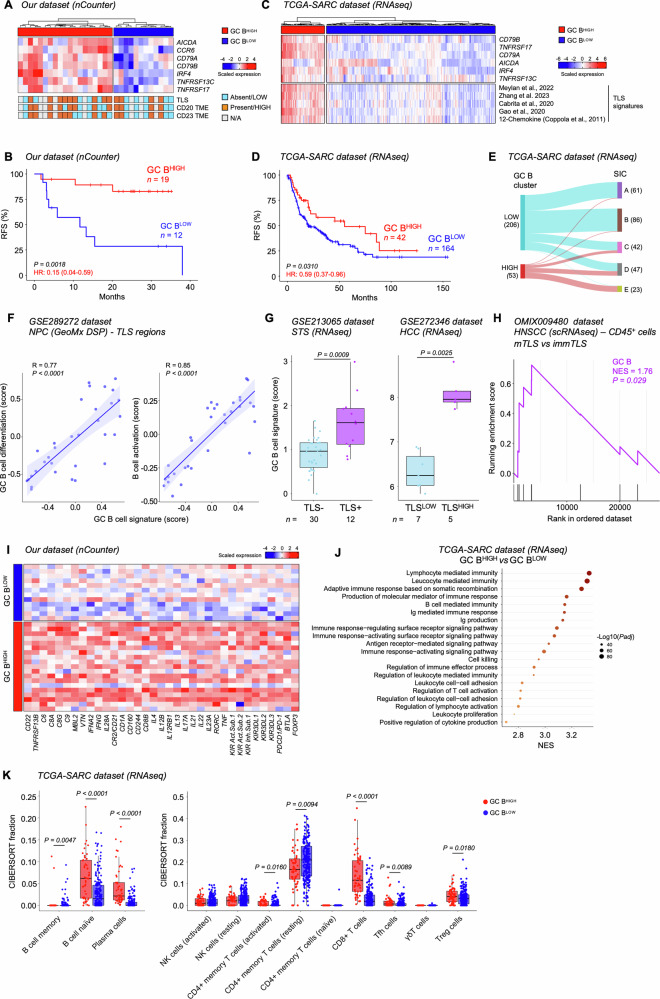
Association of a novel GC B cell signature with TLS presence and patient prognosis. **A** Hierarchical clustering of STS samples from our cohort based on the expression of GC B cell-related genes in high- (*n* = 19) and low-expressing (*n* = 12) groups. **B** Kaplan-Meier survival analysis for RFS in our patients’ cohort stratified as in (**A**). **C** Hierarchical clustering of TCGA-SARC samples (*n* = 259) using our proposed GC B cell signature, and expression of previously published TLS signatures in GC B^HIGH^ and GC B^LOW^ groups. **D** Kaplan-Meier survival curve for RFS in the TCGA-SARC dataset, upon stratification of patients followed in (**C**). **E** Sankey diagram showing the association between clustering of TCGA-SARC patients based on our GC B cell signature and sarcoma immune classes (SICs). **F** Correlation between GSVA scores for our GC B cell signature and GO:0002314 and GO:0042113 signatures within TLS regions (*n* = 32) from the spatial transcriptome GSE289272 dataset of nasopharyngeal carcinoma (NPC). Pearson R (rank correlation coefficient) and *P* values are reported. **G** GC B cell signature score in TLS^-^ and TLS^+^ advanced STS and in low- and high-density TLS hepatocellular carcinoma (HCC) (GSE213065 and GSE272346 datasets). **H** Enrichment plot of genes included in our GC B cells signature in CD45^+^ cells infiltrating head and neck squamous cell carcinoma (HNSCC) with mTLS compared to those with immTLS, from the scRNA-seq OMIX009480 dataset. NES, normalized enrichment score. **I** Heatmap of the most relevant upregulated genes in GC B^HIGH^ *vs* GC B^LOW^ samples in our cohort. **J** GSEA of differentially expressed genes between GC B^HIGH^ and GC B^LOW^ samples in the TCGA-SARC cohort. The size of the point is proportional to the significance level. **K** Comparison between CIBERSORT relative lymphoid cell fractions in GC B^HIGH^ and GC B^LOW^ TCGA-SARC samples. In (**A, C, I**) heatmaps show the scaled expression of each gene or signature score. HRs and 95% confidence intervals derived from Cox proportional hazard models are reported in (**B**, **D**). In (**G**, **K**) boxes show the median ± interquartile range; whiskers extend 1.5 times the interquartile range beyond the box. *n* values represent the number of patients (**B**, **D**) or tumor specimens (**E**, **G**). *P* values were calculated using a log-rank test (**B**, **D**), Pearson’s correlation test (**F**), Wilcoxon rank-sum test (**C**, **G**, **K**) and permutation test (**H**). In (**K**) only significant ( < 0.05) *P* values between GC B^HIGH^ and GC B^LOW^ samples are reported.

To further characterize the immune landscape of STS associated with TLS formation and B cell infiltration, we next investigated the expression of 579 immune-related genes by applying the NanoString nCounter technology to all 31 treatment-naïve STS samples collected for this study (Table 1). For patients who did receive RT or CT before surgery, biopsies obtained prior preoperative therapy were processed. We initially stratified samples based on the expression of relevant TLS-related genes and signatures [10, 20]. However, no significant associations with RFS were found, as determined by Log-rank and univariate Cox analyses (Table S3). Since for many of the published TLS- and B-cell related signatures (including the B lineage signature previously correlated to prolonged OS in STS [7]) many key genes were missing in the selected panel, we tested a novel gene signature composed of 7 genes involved in GC B cell development and differentiation [30]. This signature includes: known B cell-specific markers (*CD79A* and *CD79B*) [31, 32], class-switching and affinity maturation genes (*AICDA*, *TNFRSF13C/BAFFR* and *TNFRSF17*) [33–35], genes important for GC initiation (*IRF4*) [36], and encoding a co-stimulatory receptor expressed on activated B cells located in the GC light-zone (*CCR6*) [37]. Of note, gene selection was based on previous molecular characterization of tumor-infiltrating or TLS-associated B cells [21–23, 38], or on their inclusion in signatures for GC B cells and/or TLS tested in other tumors [23, 39, 40]. Based on the relative expression levels of genes listed above, the 31 samples of our cohort were clustered in two groups with high- and low-expression of GC B cell genes (Fig. 3A), and patients stratified as GC B^HIGH^ exhibited a longer RFS than GC B^LOW^ patients, determined by Kaplan-Meier survival curve and univariate Cox analysis (Fig. 3B and Table S3). When compared to IHC results, the majority (10/14, 71.4%) of samples classified as CD20^HIGH^, CD23^HIGH^, and/or TLS^+^ by IHC analysis (Figure S2D) were grouped as GC B^HIGH^, while only a minority (4/14, 28.6%) as GC B^LOW^ samples (Fig. 3A). This discrepancy between nCounter and IHC data could be due to several factors, including the intratumoral heterogeneity, the background noise due to the analysis of bulk RNA, and the fact that different samplings were processed for IHC and nCounter experiments for many patients.

To confirm the robustness of the prognostic relevance of this signature, as well as validate its reliability in identifying TLS^+^ samples, we harnessed data from 259 patients with localized STS profiled with RNA-seq (TCGA-SARC project) along with publicly available bulk tumor RNA-seq, scRNA-seq and spatial transcriptomic datasets with annotated information on TLS presence across STS and other cancer types [41–44]. Unsupervised clustering of TCGA-SARC samples based on the expression of GC B cell-related genes showed that this stratification method is powerful enough to discriminate the signature expression also in the TCGA-SARC cohort and confirmed the prognostic value of our signature. Notably, one gene from the signature was filtered out due to low expression, yet both the overall clustering and prognostic value remained consistent (Fig. 3C, D). Intriguingly, clustering based on our signature was distinct from SICs (Fig. 3E) with reportedly prognostic value [7], thus suggesting that this patient stratification may provide novel insights into the immune milieu of STS.

Remarkably, our signature positively correlated to GO signatures specific for GC B cell differentiation and formation (*i.e*., GO:0002314 and GO:0002467; Pearson’s *R* > 0.5, *P* < 0.0001; data not shown), and GC B^HIGH^ samples showed a significant (*P* < 0.0001; Wilcoxon rank-sum test) upregulation of relevant TLS-related signatures [20–24] (Fig. 3C). Confirming bulk RNAseq data, analysis of spatially resolved transcriptomics of TLS regions in nasopharyngeal carcinoma (NPC; GSE289272 dataset) specimens revealed a strong correlation between our signature and the GO signatures for GC B cell differentiation and B cell activation (Fig. 3F). Finally, our GC B cell signature was found increased in TLS^+^ specimens from patients with advanced STS (GSE213065 dataset), as well as in high density TLS (TLS^HIGH^) samples from neoadjuvant ICI-treated hepatocellular carcinoma (HCC; GSE272346 dataset) (Fig. 3G). In addition, the signature was enriched in CD45^+^ cells identified by scRNA-seq in head and neck squamous cell carcinoma (HNSCC) samples harboring mTLSs (OMIX009480 dataset) (Fig. 3H), thus validating the association between our signature and TLS presence across multiple cancer types.

### High expression of GC B cell signature is associated to an inflamed TME

Next, DGEA revealed 76 transcripts significantly upregulated in GC B^HIGH^ tumors compared to their lower counterpart (excluding those used to define the clusters; Table S4). Among these, other than B cell-related genes (*i.e., CD22* and *TNFRSF13B*), gene sets linked to the activation of both innate and adaptive immune responses, including genes related to the complement cascade (*i.e., C6, C8A, C8G, C9, MBL2* and *VTN*), genes encoding interferon family members (*i.e., IFNA2*, *IFNG* and *IL28A*), markers for conventional and follicular DCs (*i.e., CR2/CD21* and *CD1A)*, as well genes as associated to T and NK cell activation and functions (*i.e., CD160, CD244, CD8B, IFNG, IL4, IL12B, IL12RB1, IL13, IL17A, IL21, IL22, IL23A, RORC, TNF* and *KIRs*), were found over-represented in GC B^HIGH^ *vs* GC B^LOW^ tumors. Noteworthy, transcripts encoding co-inhibitory molecules (*i.e., PDCD1/PD-1* and *BTLA*) and the master transcription factor in regulating Treg cell development and function (*i.e., FOXP3*) were also upregulated in the GC B^HIGH^ cluster (Fig. 3I). In line with these results, GSEA showed an upregulation of several GO pathways related to lymphocyte activation, B cell-mediated immunity and immunoglobulin production, in TCGA-SARC GC B^HIGH^ samples (Fig. 3J). These findings were further confirmed by applying the CIBERSORT deconvolution algorithm [16] to estimate the relative abundance of lymphoid cell populations (Fig. 3K).

Overall, these findings suggest that an enrichment of GC B cell-signature, associated with better prognosis, is accompanied by an inflamed TME in STS.

### Th17-like cells are associated with GC formation in STS and improved prognosis

**Fig. 4:**
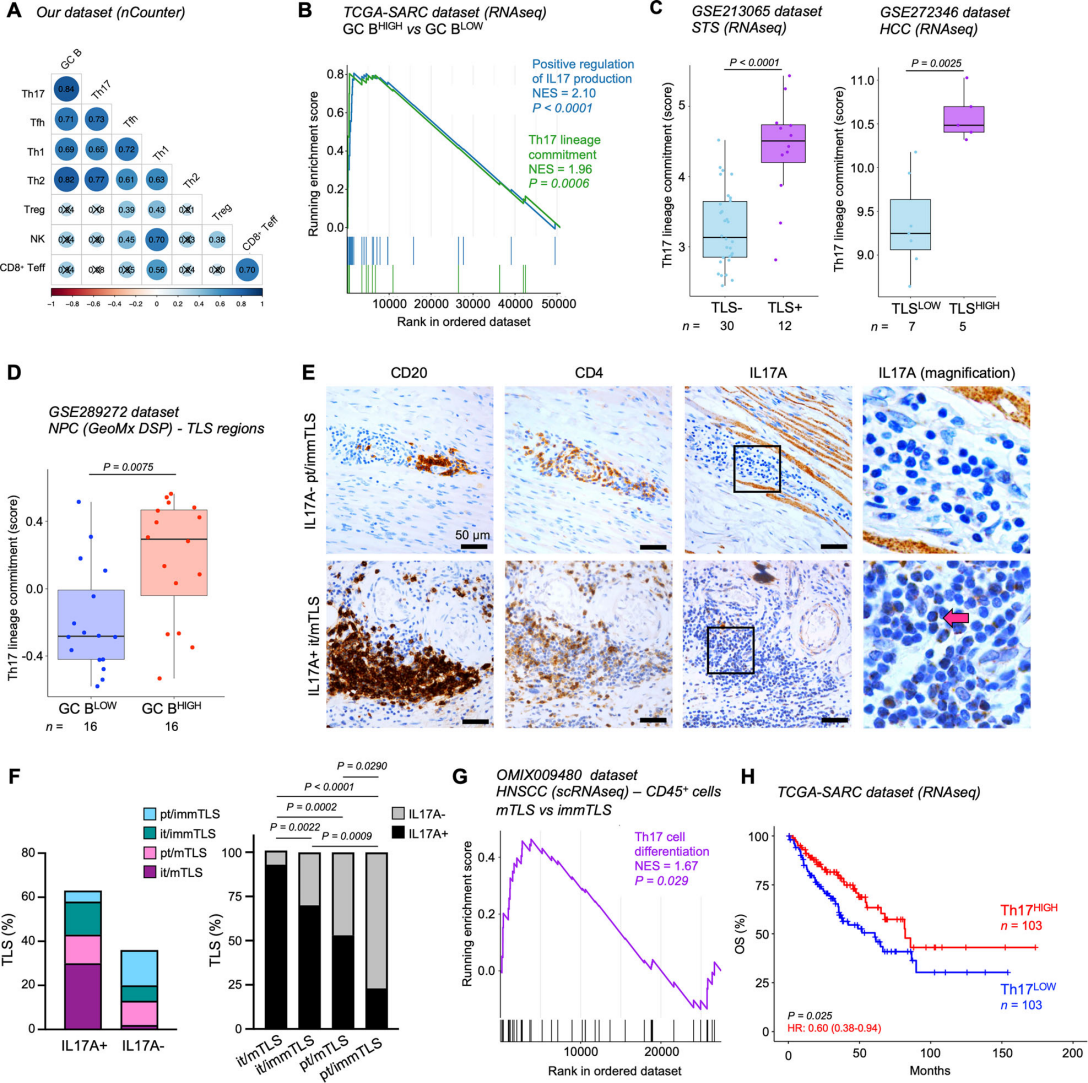
Association of Th17-like cells with GC formation and patient prognosis. **A** Correlation matrix between gene signatures specific for the indicated cell populations in samples from the nCounter cohort. Pearson’s rank correlation coefficient values (*R*) are reported. Not significant (*P* > 0.05) correlations have been crossed out. **B** Enrichment plots of gene sets involved in GO:0032740 and GO:0072540 signatures in GC B^HIGH^ compared to GC B^LOW^ tumors from the TCGA-SARC dataset. NES, normalized enrichment score. **C** Expression of the GO:0072540 signature in TLS^-^ and TLS^+^ advanced STS (GSE213065 dataset) and in low- and high-density TLS HCC (GSE272346 dataset). **D** GSVA score for the GO:0072540 signature in NPC TLS regions stratified by median score of our GC B cell signature in high- and low-expressing groups (GSE289272 dataset). **E** Representative images showing the expression of CD20, CD4 and IL17A in TLSs with different location and maturation status. The pink arrow indicates a cell showing clear IL17A positivity and lymphoid morphology features. Scale bars: 50 µm. **F** Quantification of TLS with different maturation status and location containing at least one cell positive for IL17A staining and showing clear lymphoid morphology features, determined by IHC. A total of 123 TLSs from 10 TLS^+^ samples were included in this analysis. **G** Enrichment plot of genes included in the GO:0072539 signature in CD45^+^ cells infiltrating HNSCC with mTLSs compared to those with immTLSs (OMIX009480 dataset). **H** Kaplan-Meier survival analysis of patients’ OS generated for the GO:0072540 signature in the TCGA-SARC dataset. Patients were stratified by median values of single-sample scores. HR and 95% confidence interval derived from Cox proportional hazard models are reported. In (**C**, **D**) boxes show the median ± interquartile range; whiskers extend 1.5 times the interquartile range beyond the box. *n* values represent the number of tumor specimens (**C**), TLS regions (**D**) or patients (**H**)*. P* values were calculated using Pearson’s correlation test (**A**), permutation test (**B**, **G**), Wilcoxon rank-sum test (**C**, **D**), Fisher’s exact test (**F**), and log-rank test (**H**). Only significant ( < 0.05) values are shown in (**F**).

To identify inflammatory mechanisms associated to TLS formation in STS, we next searched for gene signatures discriminating for specific lymphoid subtypes (Table S1) positively correlated to our GC B cell signature in our cohort. A positive correlation was identified with Tfh-, Th1-, Th2- and Th17-related signatures (Fig. 4A), in line with the over-representation of genes encoding key cytokines and transcription factors specific for these phenotypes in GC B^HIGH^ samples (Fig. 3I). Among these, the strongest correlation was observed between GC B cell- and Th17 cell-related signatures (Fig. 4A). To validate a potential correlation between TLS presence, GC formation and Th17-like cells we first leveraged publicly available in silico data. Consistent with our findings, we observed a positive enrichment of GO signatures indicative of “Th17 lineage commitment” and “positive regulation of IL17 production” in GC B^HIGH^ TCGA-SARC samples (Fig. 4B). Similarly, TLS^+^ advanced STS samples exhibited higher expression of Th17-related genes compared to their negative counterpart (Fig. 4C). A similar increase was also confirmed in TLS^HIGH^ HCC samples and in GC B^HIGH^ TLS regions from NPC tumors (Fig. 4C, D).

Next, to probe a potential causative link between Th17-like cells and STS-associated TLSs we performed IHC staining with an anti-IL17A antibody. Both tumor and immune cells showed cytoplasmic staining for IL17A (Figure S3A). Of note, no significant differences were observed in terms of IL17A expression by tumor cells when we compared TLS^+^ and TLS^-^ specimens, nor between specimens obtained from relapse *vs* non-relapsed patients (Figure S3B). Although in some samples the quantification of IL17 positivity on isolated intratumoral immune cells and in some TLSs was hampered by the elevated background, we were able to evaluate IL17A expression in most TLSs identified by CD3/CD20 staining (Fig. 1). Notably, the majority of immune cells infiltrating TLSs and expressing IL17A exhibited classical lymphoid morphological features (*i.e*., small nucleus, scant cytoplasm, high nucleus-to-cytoplasm ratio), while only a few cells displayed morphologic features of DCs or macrophages (Figure S3A). IHC staining for CD4 and IL17A on adjacent tissue sections revealed a high concordance between CD4 and IL17 positivity and allowed the identification of cells displaying both cytoplasmic IL17A and membranous CD4 staining, consistent with a Th17-like cell phenotype (Figure S3C). Interestingly, Th17-like cells were found in the majority (78/123; 63.4%) of TLSs, and among IL17A^+^ TLSs the majority were mTLS located intratumorally (Fig. 4E, F). Confirming our findings, the GO transcriptional signature associated with “Th17 cell differentiation” was found significantly enriched in CD45^+^ cells infiltrating HNSCC tumors with mTLSs, as compared to those with immTLSs (Fig. 4G).

Finally, analysis of the TCGA-SARC dataset revealed that a high Th17-related gene signature marked a favorable OS outcome in STS patients (Fig. 4H). Overall, these data suggest a correlation between the presence of Th17-like cells and TLS formation and maturation, and identified a prognostic value for Th17 transcriptional signatures in STS.

### Immunosuppressive M2-like macrophages infiltrate the TME of GC B^LOW^ STSs and mostly reside in immTLSs

**Fig. 5:**
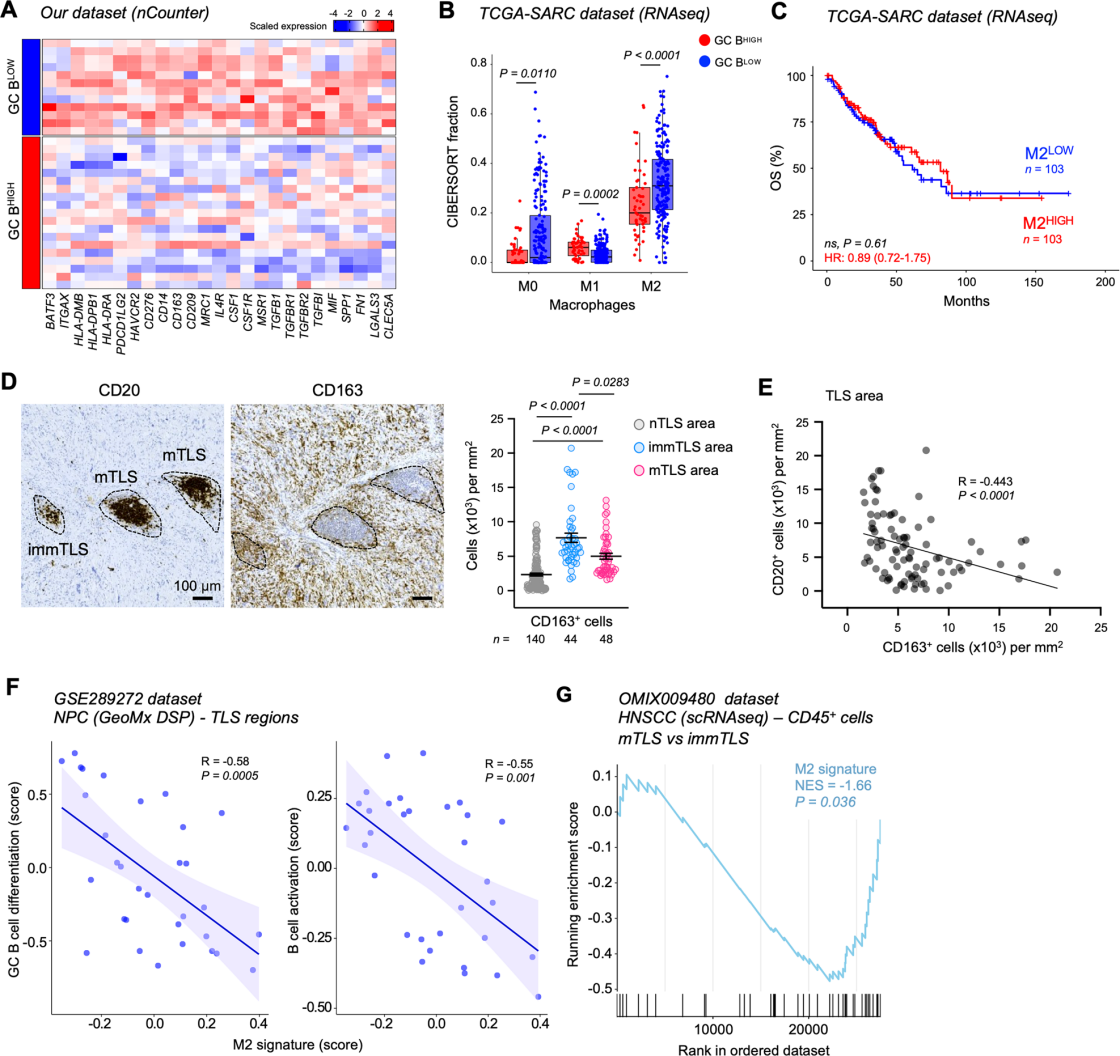
Association of M2-like macrophages with an immTLS status. **A** Heatmap of the most relevant upregulated genes in GC B^LOW^ compared to GC B^HIGH^ samples in our nCounter dataset. **B** CIBERSORT macrophage fractions in GC B^LOW^ and GC B^HIGH^ TCGA-SARC samples. Boxes show the median ± interquartile range; whiskers extend 1.5 times the interquartile range beyond the box. **C** Kaplan-Meier survival curve for OS of TCGA-SARC patients stratified by median CIBERSORT fraction for M2 macrophages. **D** Representative images of CD20 and CD163 expression and quantification of CD163^+^ cell density (count/mm^2^) in nTLS, mTLS and immTLS areas. Each dot denotes one individual immTLS, mTLS or round ROI from nTLS areas obtained from *n* = 13 TLS^+^ samples. Mean ± SEM is shown. Scale bars: 100 µm. Representative image for CD20 staining is the same showed in Fig. 2A. **E** Correlation between CD163^+^ and CD20^+^ cell densities within TLS (*n* = 92). Spearman R (rank correlation coefficient) and *P* values are reported. **F** Correlation between GSVA scores for the M2 signature [25], GO:0002314 and GO:0042113 signatures, within NPC TLS regions (*n* = 32) (GSE289272 dataset). Pearson R (rank correlation coefficient) and *P* values are reported. **G** Enrichment plot of genes included in the M2 signature in CD45^+^ cells infiltrating HNSCC with mTLS compared to those with immTLS (OMIX009480 dataset). *n* values represent the number of patients (**C**) or individual nTLS or TLS area (**D**). HRs and 95% CI derived from Cox proportional hazard models are reported in (**C**). *P* values were calculated using Wilcoxon rank-sum test (**B**), log-rank test (**C**), Kruskal-Wallis with uncorrected Dunn’s test (**D**), Spearman’s rank correlation test (**E**), Pearson’s rank correlation test (**F**) and permutation test (**G**).

We next interrogated our nCounter data to identify immune signatures linked to reduced GC B cell organization in STSs. Although GC B^LOW^ tumors showed increased levels of genes involved in antigen presentation, including markers for conventional type 1 DCs (*i.e., BATF3* and *ITGAX*/*CD11c*) and genes encoding MHC class II molecules (*i.e., HLA-DMB*, *HLA-DPB1* and *HLA-DRA*), low expression of GC B signature was coupled to the upregulation of genes encoding inhibitory checkpoint molecules, such as *PDCD1LG2* (encoding PD-L2), *HAVCR2* (better known as TIM3) and *CD276* (also known as B7-H3) (Fig. 5A and Table S4), which expression (by cancer or immune cells) has been linked to immunosuppression in several cancer types [45–47]. Additionally, DGEA showed an over-representation of multiple well-known marker genes for tumor-associated macrophages (TAMs) characterized by an immunosuppressive phenotype (also referred as to “M2-like” [48]) in GC B^LOW^ *vs* GC B^HIGH^ tumors, including the upregulation of *CD14* (encoding one of the main differentiation markers of myeloid lineage), *CD163, CD209*, *MRC1* (also known as CD206) and *IL4R* (Fig. 5A). Among other upregulated genes underlying immunosuppressive and pro-tumorigenic properties of TAMs, we found: genes encoding the colony stimulating factor 1 (*CSF1*) and its receptor (*CSF1R*), which targeting reportedly reduced M2 polarization and improved disease control in various preclinical settings [48]; *MSR1*, encoding a scavenger receptor that mediates the TAM polarization toward a M2-oriented phenotype [49]; genes involved in transforming growth factor β (TGF-β) signaling, which role in promoting tumor progression and invasion has been intensively studied [50]; as well as *MIF* (encoding the macrophage migration inhibitory factor) and genes associated to secreted phosphoprotein 1-positive (SPP1^+^) TAMs (*i.e., SPP1*, *FN1*, *LGALS3* and *CLEC5A*), that together have been correlated to tumor progression in a preclinical model of STS [51] (Fig. 5A). Corroborating these findings, GC B^LOW^ TCGA-SARC samples exhibited significantly higher CIBERSORT fraction of M2 macrophages and increased *SPP1* expression, paralleled by reduced fraction of M1 TAMs and decreased expression of *TIMD4* (better known as *TIM4*) and *FOLR2* (Fig. 5B and S4A), whose expression by TAMs has been linked to TLS formation in different cancer settings [52–54].

To gain better insights on the spatial relationships between TAMs and TLSs, we performed the IHC staining of CD163. At odds, we observed no significant differences in CD163^+^ cell accumulation in the whole TME among STS specimens divided based on TLS presence or CD20^+^ B cell accumulation, while a higher accumulation of CD163^+^ macrophages was observed in pleomorphic sarcomas (Figure S4B), even if they showed a higher TLS incidence and density compared to other histotypes (Fig. 1G). In line with these data, stratification of samples based on median CD163^+^ cell density in the TME showed no impact on STS survival (Figure S4C). Similarly, no correlation was found between the CIBERSORT fraction of M2-like macrophages and STS prognosis in the TCGA-SARC cohort (Fig. 5C).

We next focused our analysis on CD163^+^ cells infiltrating TLSs with different maturation status. Similarly to the other investigated lymphoid populations (Fig. 2D), CD163^+^ cells were mostly located within TLS areas as compared to non-TLS areas (Fig. 5D). Intriguingly, immTLSs were highly infiltrated by CD163^+^ macrophages compared to mTLSs (Fig. 5D), and while IHC revealed no significant correlations between CD163^+^ and CD20^+^ B cell densities in the entire TME (Spearman’s *R* = 0.12, *P* = 0.63; data not shown), we observed a negative correlation between accumulation of CD163^+^ and CD20^+^ cells within TLS areas (Fig. 5E)

In summary, these findings suggest an association between M2-like macrophages and TLSs characterized by an immature phenotype. Further supporting our hypothesis, a M2-related signature [25] negatively correlated with GO signatures for “GC B cell differentiation” and “B cell activation” in NPC-associated TLSs, and was negatively enriched in CD45^+^ cells infiltrating HNSCC tumors with mTLSs (Fig. 5F and G).

### Integration of gene signatures for GC B cells, Th17 cells and M2 macrophages and their association to STS survival

**Fig. 6:**
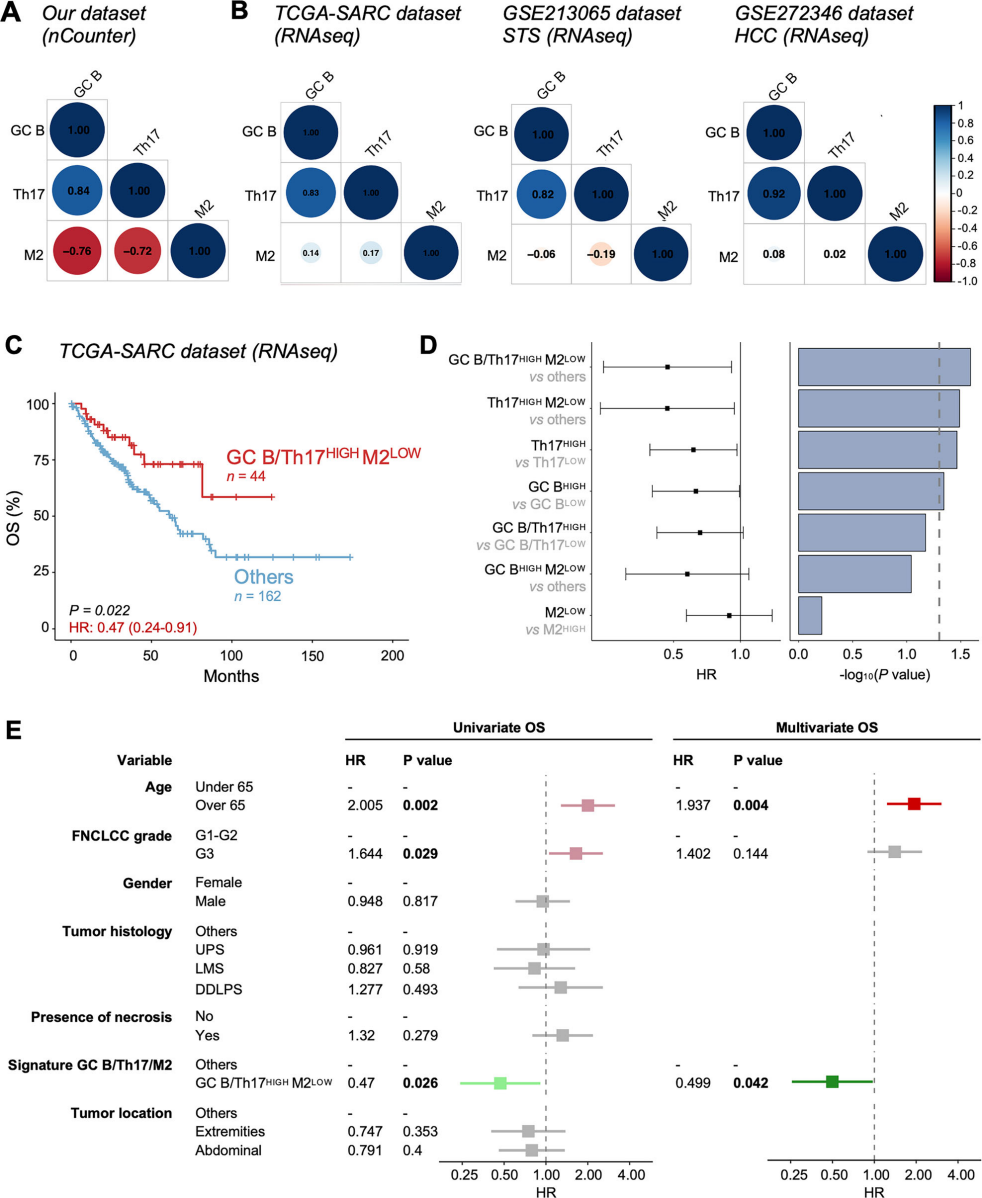
Prognostic impact of a transcriptomic signature discriminating GC B cells, Th17 cells and M2 macrophages in STS. **A**, **B** Correlation matrix between gene signatures specific for the indicated cell populations in samples from our nCounter cohort and the indicated RNA-seq datasets. Pearson’s rank correlation coefficient values are reported. **C** Kaplan-Meier survival curve showing OS of TCGA-SARC patients stratified by integrating gene expression signatures of GC B cells (GO:0002314), Th17 cells (GO:0072540) and CIBERSORT fraction of M2 macrophages. Number of patients and P values (by log-rank test) are reported. **D** Comparative analysis of the prognostic value of the integrated GC-B/Th17/M2 signature versus individual and partial signatures in TCGA-SARC patients related to OS. The forest plot (*left)* displays HRs and 95% confidence intervals. The bar plot (*right*) shows the significance of each HR. The dotted vertical line indicates the significance threshold (*P* = 0.05). **E** Univariable and multivariable Cox proportional hazard regression of the integrated signature in relation to OS in TCGA-SARC patients. Significant univariate and multivariate HRs are shown in red (HR > 1) and green (HR < 1). HRs and 95% CI derived from Cox proportional hazard models are reported in (**C**, **E**).

Since TAM impact on cancer prognosis is highly context-dependent and varies according to the TME composition [48], we next investigated their potential relationship with GC B cells and Th17-like cells in STS samples. nCounter data revealed a strong inverse correlation between TAM-associated genes and GC B cell- and Th17 cell-related signatures, while a slight correlation was observed between the CIBERSORT fraction of M2 macrophages and GO signatures discriminating GC B cells and Th17 cells in the TCGA-SARC dataset and in samples from advanced STS patients (Fig. 6A and B). These findings were independently confirmed in the HCC dataset (Fig. 6B).

The clinical implication of these three populations was assessed upon stratification of TCGA-SARC patients by integrating M2-, Th17- and GC B-related signatures. Interestingly, a lower CIBERSORT fraction of M2 macrophages together with the co-enrichment of GC B- and Th17-related GO signatures was associated with the best survival outcome (considered as OS), significantly outperforming the three signatures used alone or the combination of just two signatures (Fig. 6C, D). Finally, the survival association between the GC B/Th17^HIGH^M2^LOW^ group and OS was confirmed by a univariate and multivariate Cox proportional hazard regression model, adjusting for relevant clinical parameters, such as age, gender, tumor’s grade, location, histotype and presence of necrosis (Fig. 6E).

In conclusion, we here refined a novel gene expression signature that specifically reflects the cellular composition of TLS with GC structures and accurately predicts STS survival outcomes.

## Discussion

TLSs and B cells have been associated with favorable disease outcomes and improved responses to immunotherapy across multiple human cancers [10], including STS [7, 9]. However, information regarding TLS development and involvement in antitumor immunity in STS remains limited. Herein, we harnessed IHC and transcriptomic profiling of STS specimens to gain deeper insights into the immunological landscape of STS-associated TLSs, and validated our findings by leveraging publicly available bulk, single-cell, and spatial RNA-seq datasets with annotated information regarding TLS presence and maturation status. We observed that TLSs are highly heterogeneous in terms of amount, location, and maturation status among STS specimens, and identified a novel GC B cell gene signature associated with TLS presence, enhanced immune activation, and improved clinical outcomes in STS. Notably, our proposed signature stratified TCGA-SARC patients differently from well-known immune classes with reported prognostic relevance [7], thereby revealing aspects of TLS biology and humoral immunity that are not reflected in the SIC-based classification. Specifically, we found that tumor with TLSs and exhibiting high expression of GC B cell-related gens showed an upregulation of Th17-related signatures, accompanied by the downregulation of genes associated to M2-like TAMs. Moreover, IHC showed distinct spatial distribution patterns of these two cell subsets, with Th17-like cells mostly localized within mTLSs, whereas CD163^+^ M2-like macrophages mainly infiltrated immTLSs. Importantly, patient stratification based on combined signatures for GC B cells, Th17 cells and M2 macrophages demonstrated a higher prognostic value compared with stratification based solely on GC B cell presence.

Given the profound impact of TLSs on antitumor immunity, an increasing number of reports have investigated the mechanisms underlying TLS neogenesis, with the aim to support the development of novel therapeutic strategies to boost ICI efficacy [11]. However, in the context of STS, studies exploring cellular and molecular mechanisms favoring or hampering B cell organization in TLSs are still lacking, and the induction of TLS neogenesis in STS patients lacking these structures remains a challenging opportunity. In this study, we identified for the first time two immune cell subsets with potentially opposing roles in TLS formation and maturation: Th17-like cells and M2-like macrophages.

Although the role of IL17 and Th17 cells in cancer remains highly controversial [55–60], accumulating evidence suggests that Th17 cells might act as lymphoid tissue inducer (LTi) by initiating TLS formation, thus supporting our hypothesis that Th17-like cells could serve as key mediators of TLS development and functionality in STS. For instance, IL17 production by Th17 cells induced TLS development in chronic inflamed tissues by promoting the CXCL13-dependent recruitment of B cells by stromal cells [61–63], and spatial transcriptomics of TLSs in gastric cancers showed a correlation between signatures for B cell receptor signaling and Th17 differentiation [64]. Similarly, upregulation of Th17 cell-related genes was found in PDAC tumors containing TLSs, as well as in microdissected lymphoid aggregates from PDAC specimens harvested after allogeneic vaccine [65, 66]. Corroborating these finding, recent spatial profiling of human glioma-associated TLSs showed that Th17-like cells expressing markers of LTi functions represent up to 50% of all T cells forming the TLS [67], and adoptively transferred Th17-polarized cells promoted long-lasting B cell-mediated antitumor immunity, upon IL21 secretion and CD40L-dependent co-stimulation, in a preclinical model of melanoma [68].

In contrast to Th17-like cells, our findings suggest an association between CD163^+^ M2-like macrophages and an immTLS status. Supporting our data, increased infiltration of CD163^+^ TAMs in immTLS was observed by IHC in specimens of clear cell renal cell carcinoma and metastatic gastric cancer [64, 69]. Furthermore, scRNA-seq revealed a higher abundance of M2-like macrophages expressing metalloproteinases 9 and 12 in HNSCC samples with immTLSs compared to those with mTLSs [43].

TAMs are a major component of the TME in multiple human cancers, including STS [70], and depleting TAMs have emerged as a promising therapeutic approach to promote antitumor immune responses [48]. However, TAM depletion with pan-macrophage depleting antibodies used as monotherapy (*e.g*., anti-CSF1R antibodies) has shown limited efficacy both in humans and mice [48], including a mouse model of undifferentiated pleomorphic sarcoma [51], thus calling for a better understanding of TAM phenotypes and interactions with other components of the TME for the identification of more efficient strategies. Although our results have not defined the precise TAM phenotype associated to an immTLS status in STS, among M2-related genes deregulated in GC B^LOW^ tumors we find intriguing the upregulation of *MIF* and of genes linked to a pro-tumorigenic and extracellular matrix (ECM)-remodeling phenotype of SPP1^+^ TAMs, for the following reasons: (i) *MIF* depletion limited tumor growth by reducing the intratumoral accumulation of SPP1^+^ TAMs in a preclinical STS model [51]; (ii) SPP1^+^ TAMs are known to drive CD8^+^ T cell exhaustion, tumor progression and treatment resistance in various cancer types [71]; (iii) multi-omic profiling of non-small cell lung cancers non-responding to neoadjuvant chemo-immunotherapy recently showed a correlation between SPP1^+^ TAMs, cancer-associated fibroblasts (CAFs) and reduced TLS signature, thus suggesting that SPP1^+^ TAM-CAF interactions might hamper TLS formation [72]. Future studies should be pursued to elucidate the spatial association and implication of SPP1^+^ macrophages with STS-associated TLSs.

It is important to acknowledge the limitations of our study to avoid unwarranted overinterpretation. First, our sample size was constrained by the rarity of STS and highly heterogeneous, as we included multiple sarcoma histologies in our cohorts. However, this composition reflects most of the publicly available datasets, some of which were also used to validate the findings obtained in our cohort. Furthermore, given that different STS histotypes are known to exhibit distinct prognosis and ICIs response, histology was included as a covariate in our multivariable models. Second, although our nCounter data were validated by leveraging a larger cohort of STS specimens (*i.e*., the TCGA-SARC dataset) along with other four publicly available bulk, single-cell and spatial transcriptomic datasets containing information regarding TLS presence and maturation status in STS and other cancer types (*i.e*., NPC, HCC and HNSCC), the impact of TLS localization and maturation heterogeneities on divergent clinical outcomes in STS will need to be further elucidated in larger patient cohorts. Third, although our study identified an association between Th17-like cells and M2-like macrophages with TLS of different maturation status, further in-depth phenotypic and spatial characterization of these cell subsets is warranted, followed by mechanistic investigations, to refine the complex cell-to-cell interaction networks within TLS.

Although the cellular dynamics underlying TLS-mediated immunity continue to elude our complete understanding, our study gained novel insights on cellular features associated to TLS maturation and improved prognosis in adult STS, thus providing a foundation for future research efforts aiming at the development of therapeutic interventions for the induction or maturation of TLS.

## Availability of data and material

All data generated in this study are included in this article and its supplementary information. Normalized nCounter expression data generated for this study are reported in Table S5. Any additional information required to re-analyze the data reported in this work paper is available from the corresponding author upon reasonable request. Public transcriptomics datasets that were used in this study can be found on the GDC Data Portal (portal.gdc.cancer.gov) as project TCGA-SARC and on the Gene Accession Omnibus (ncbi.nlm.nih.gov/geo/) as GSE213065, GSE272346, GSE289272 and on the OMIX database (ngdc.cncb.ac.cn/omix/) under accession code OMIX009480.

## Supplementary information

Supplementary figures S1-S4

Supplementary Table 1

Supplementary Table 2

Supplementary Table 3

Supplementary Table 4

Supplementary Table 5
