# Supplementary material for: Th17-like cells and immunosuppressive macrophages infiltrate tertiary lymphoid structures with distinct maturation status in soft-tissue sarcoma
Source: Cell Death Dis. 2025 Dec 22;16(1):917. doi: 10.1038/s41419-025-08376-4 (PMC12748978; doi:10.1038/s41419-025-08376-4)
Supplement: Supplementary file 1 — Supplementary figures S1-S4 [file 41419_2025_8376_MOESM1_ESM.pdf]

Supplementary figures

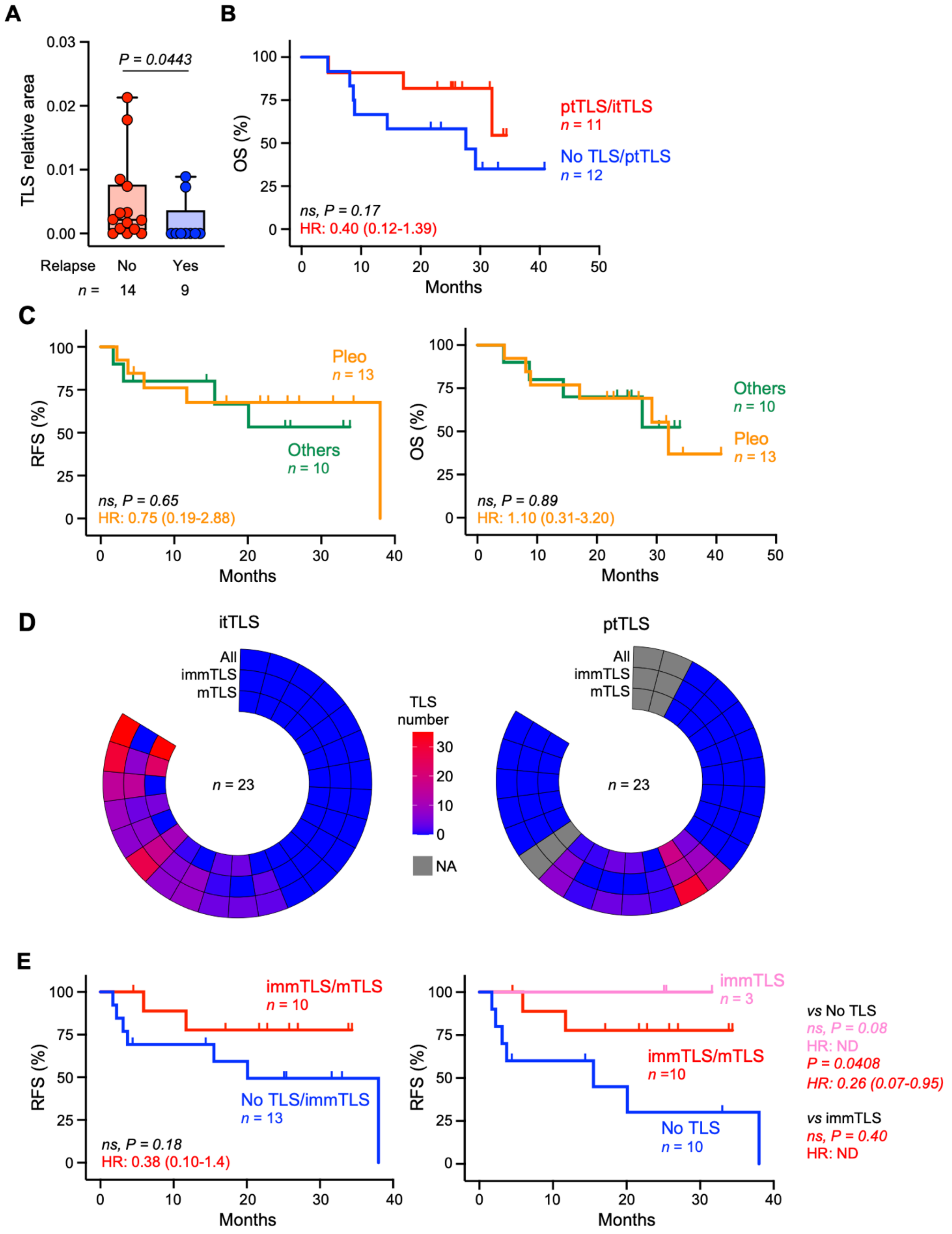

Figure S1. Impact of different histological subtypes and TLS maturation on survival. A) Box plot showing tertiary lymphoid structure (TLS) relative area (expressed as the ratio between the area of all TLSs

and the tissue area considered for the analysis) in non-relapsed and relapsed soft-tissue sarcoma (STS) patients. Each dot denotes one tissue sample. **B**) Kaplan-Meier curve of overall survival (OS) among STS patients with no TLS or with only peritumoral (pt) TLS (no TLS/ptTLS group) and patients harboring at least one intratumoral (it) TLS (ptTLS/itTLS group). **C**) Kaplan-Meier curves of patients with pleomorphic sarcomas (pleo) and other STS histotypes for relapse-free survival (RFS) and OS. **D**) Distribution of mature (m) and immature (imm) TLSs in tumor and peritumor compartments, across 23 STS patients. Samples' order is the same shown in Figures 1C, 1D and 2C. **E**) RFS curves of STS patients with at least one mTLS (imm/mTLS group) compared to patients with no TLS or/and only immTLS patients (left panel: no TLS/immTLS group; right panel: immTLS group; noT LS group). Hazard Ratio (HR) and 95% confidence interval calculated by log-rank method are reported in (**B, C, E**). *n* values represent the number of individual tumors (**A, D**) or patients (**B, C, E**). *P* values were calculated using unpaired Mann-Whitney test (**A**), and Log-Rank test (**B, C, E**).

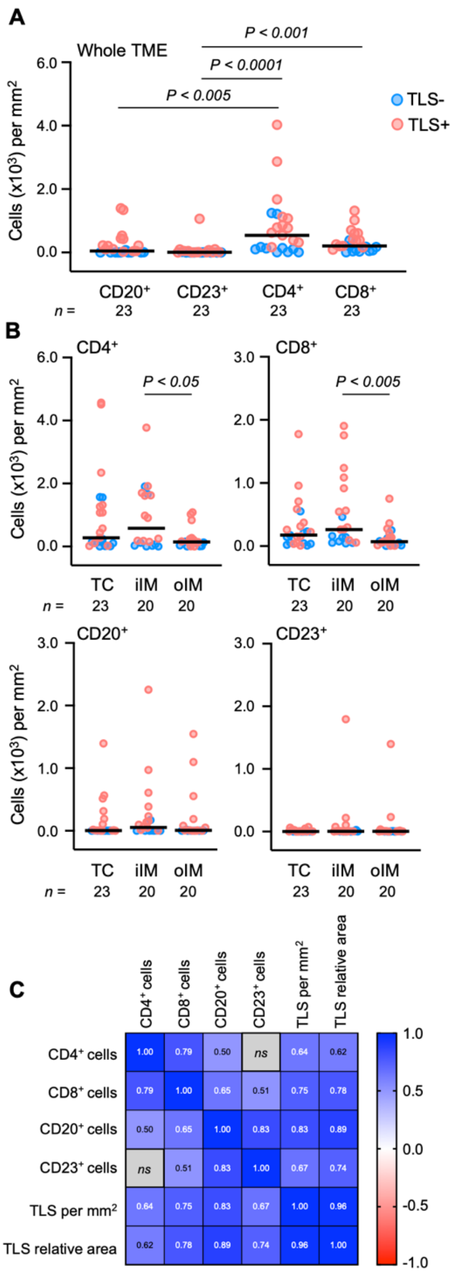

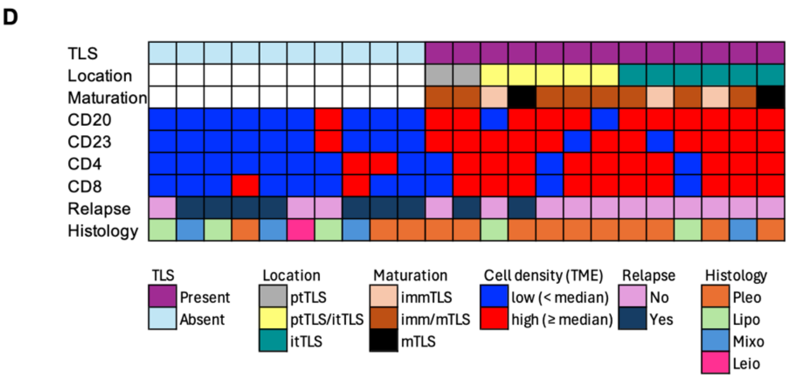

E

	Group	N	RFS *P* value	RFS HR (CI)	RFS Median RFS	OS *P* value	OS HR (CI)	OS Median OS
CD20	LOW	11	-	-	20.1	-	-	17.1
	HIGH	12	*0.0334*	0.24 (0.06-0.89)	NR	*0.0341*	0.26 (0.07-0.94)	NR
CD23	LOW	11	-	-	20.1	-	-	27.6
	HIGH	12	*0.0406*	0.24 (0.07-0.91)	NR	*ns, 0.15*	0.41 (0.11-1.47)	NR
CD4	LOW	11	-	-	38	-	-	27.6
	HIGH	12	*ns, 0.74*	0.81 (0.22-3.01)	NR	*ns, 0.83*	0.87 (0.25-3.02)	32
CD8	LOW	11	-	-	NR	-	-	27.6
	HIGH	12	*ns, 0.21*	0.46 (0.12-1.77)	38	*ns, 0.19*	0.44 (0.13-1.58)	32

Figure S2. Cellular composition of the TME in STS. A, B) Density of CD4$^+$ cells, CD8$^+$ cells, CD20$^+$ cells and CD23$^+$ cells in the entire TME (**A**) and in the indicated tissue regions (**B**) across 23 STS samples. Each dot denotes one tissue sample and median values are shown. In (**A**) individual values are expressed as the mean of 12-to-24 round ROIs from 3 tissue regions: 2 intratumoral regions, divided in tumor center (TC) and inner invasive margin (iIM; tumor area within 1 mm from the tumor front), and 1 peritumoral region corresponding only to the tissue surrounding the tumor nest within 1 mm of the tumor front (referred as to outer IM; oIM). In (**B**) individual values are expressed as the mean of 4-to-8 round ROIs from each tissue region. **C**) Correlation matrix between the indicated cell densities in the entire TME, TLS density and relative area. Spearman's rank correlation coefficient values (R) for significant ($P < 0.05$) correlations are reported. **D**) Associations between

TLS presence, TLS location, TLS maturation, density of $CD4^+$ cells, $CD8^+$ cells, $CD20^+$ cells and $CD23^+$ cells in the entire TME, relapse status (evaluated as local or distant disease recurrence observed after surgical resection) and histological subtypes (Leio, leiomiosarcoma; Lipo, liposarcoma; Myxo, mixofibrosarcoma; Pleo, pleomorphic sarcoma) in 23 STS patients. **E**) Log-rank test results for RFS and OS upon stratification of 23 STS patients based on median $CD20^+$, $CD23^+$, $CD4^+$ or $CD8^+$ cell abundance in the whole TME in high- (≥ median) and low-expressing groups (< median). CI, 95% confidence interval; HR, hazard ratio; NR: not reached. *n* values represent the number of individual tumors (**A, B**) or patients (**E**). *P* values were calculated using paired Fridman with uncorrected Dunn's test (**A, B**), Spearman's rank correlation test (**C**), and log-rank test (**E**). In (**A, B**) only significant (< 0.05) *P* values are shown.

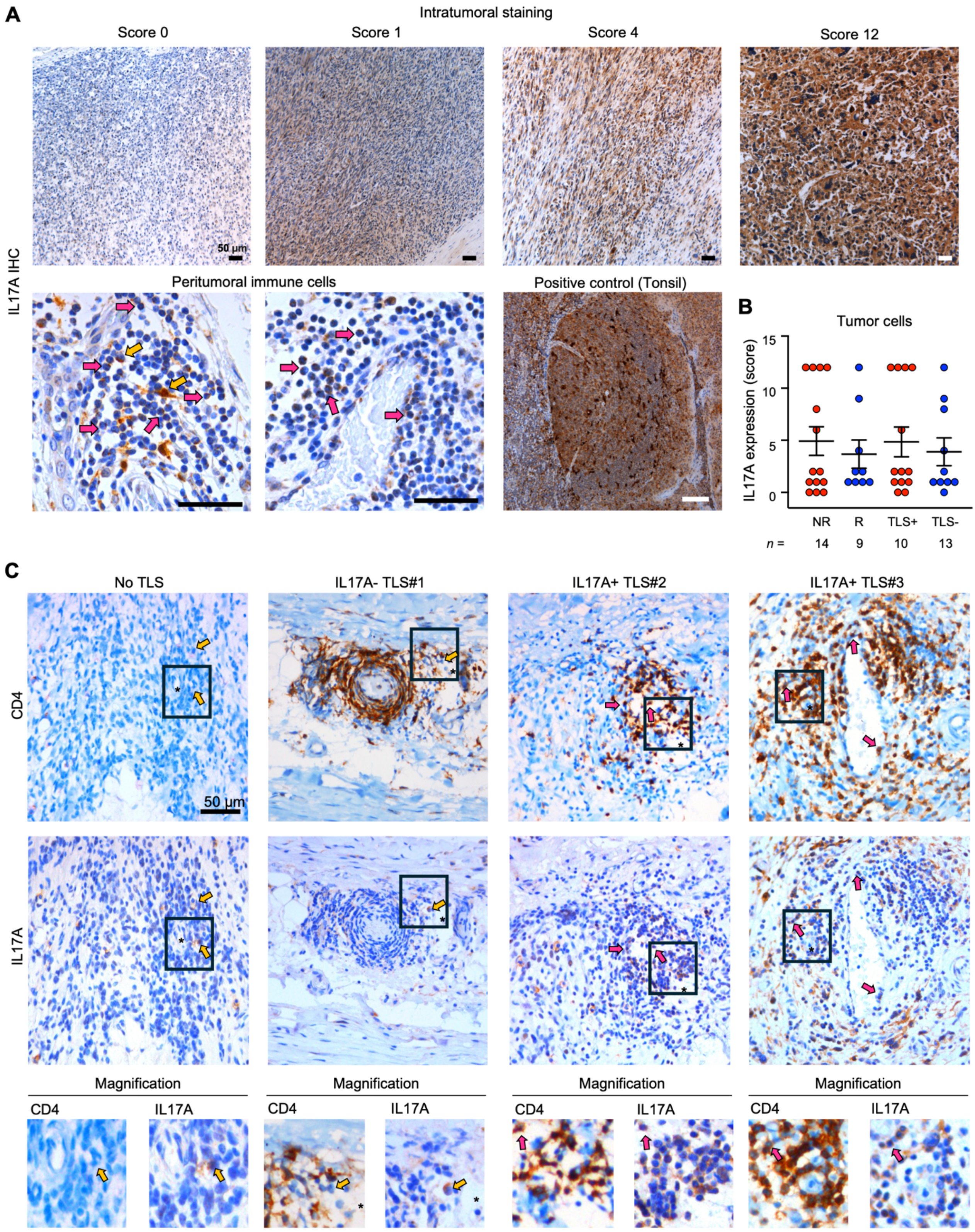

Figure S3. IL17A expression in STS samples. A) Representative images showing IL17A expression by tumor and peritumoral immune cells. Images for different tumoral scores are shown. Pink arrows indicate $IL17A^{+}$ immune cells with typical morphological features of lymphoid cells, the yellow arrows indicate $IL17A^{+}$ cell with typical morphological features of dendritic cells or macrophages. A representative image

from a positive control (tonsil) is also included. Scale bars: 50 μm. **B**) Quantification of IL17A expression by tumor cells. IL17A expression by tumor cells was scored considering the percentage of positively stained cancer cells (P) and the intensity of staining (I), and the final score is the result of multiplying these values (score = P x I). Each dot denotes one sample. Mean ± SEM is shown. *n* values represent the number of individual tumors. *P* values were calculated using unpaired Mann-Whitney test. **C**) Concordance between IL17A and CD4 positivity evaluated by single IHC stainings on adjacent 3 μm tissue sections. Representative images show IL17A and CD4 expression in an intratumoral non-TLS area, an TLS defined as IL17A-negative and two TLSs identified as IL17A-positive. Pink arrows indicate cells double positive for cytoplasmic IL17A and membranous CD4, exhibiting typical lymphoid morphological features (*i.e.,* small nucleus, scant cytoplasm, high nucleus-to-cytoplasm ratio), consistent with a Th17-like phenotype. Conversely, yellow arrows highlight IL17A-positive but CD4-negative cells characterized by a lower nucleus-to-cytoplasm ratio.

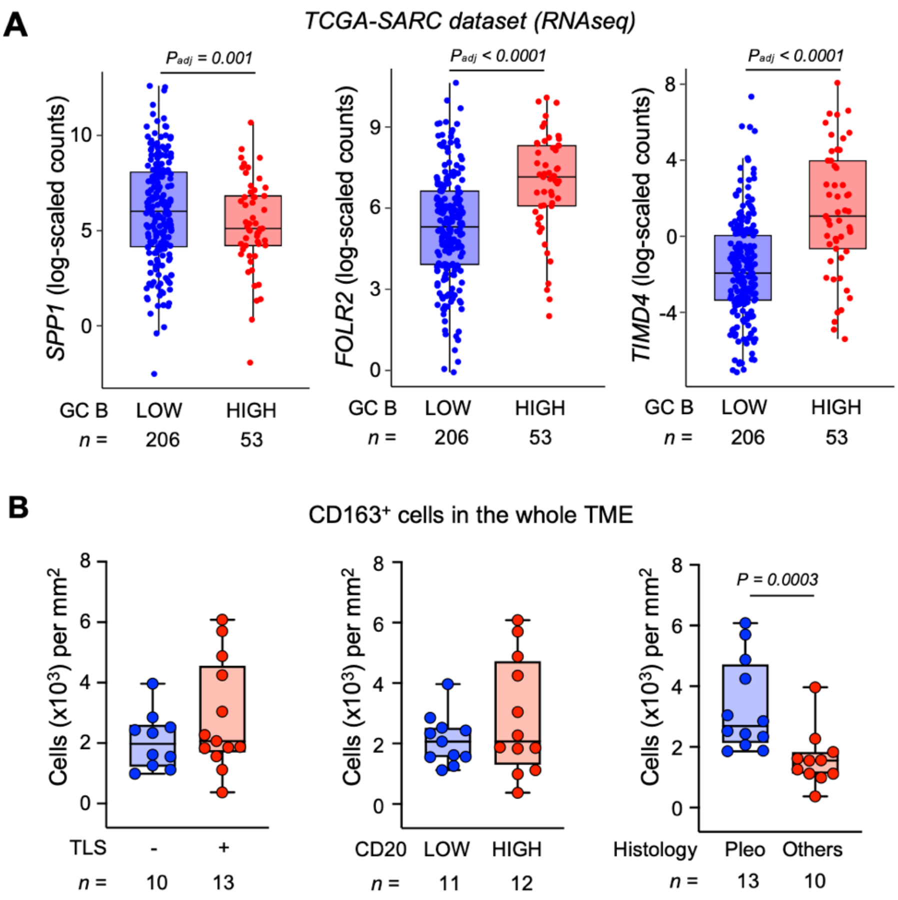

C

	Group	N	RFS			OS		
			**P value**	**HR (CI)**	**Median RFS**	**P value**	**HR (CI)**	**Median OS**
**CD163**	LOW	11	-	-	NR	-	-	32
	HIGH	12	*ns, 0.73*	1.26 (0.33-4.83)	38	*ns, 0.70*	0.79 (0.23-2.74)	35

Figure S4. Macrophage markers' expression in STS samples. A) Box plot showing the expression of *SPP1*, *FOLR2* and *TIMD4* (better known as *TIM4*) in GC B^{HIGH} and GC B^{LOW} TCGA-SARC samples. **B**) Box plots showing the density (count/mm^2) of CD163$^+$ cells in the whole TME determined by IHC and QuPath analysis in samples divided based on the presence/absence of TLSs, CD20$^+$ cell density in the TME, and histological subtype. Each dot denotes one tissue sample, and values are expressed as the mean of 12-to-24 round ROIs for each sample. Boxes show the median ± interquartile range; whiskers indicate minima and maxima. **C**) Log-rank test results for RFS and OS upon stratification of 23 STS patients based on median CD163$^+$ cell abundance in the whole TME in high- (≥ median) and low-expressing groups (< median). CI, 95% confidence interval; HR, hazard ratio; NR: not reached. *n* values represent the number of individual tumors (**A, B**) or patients (**C**).

P values were calculated using a Wald test (**A**), unpaired Mann-Whitney test (**B**), and log-rank test (**C**). In (**B**) only significant (< 0.05) *P* values are shown.
